# Contribution to the molecular systematics of the genus *Capoeta *from the south Caspian Sea basin using mitochondrial cytochrome b sequences (Teleostei: Cyprinidae)

**Published:** 2016-06

**Authors:** Halimeh Zareian, Hamid Reza Esmaeili, Adeleh Heidari, Majid Reza Khoshkholgh, Hamed Mousavi-Sabet

**Affiliations:** 1 Department of Biology, College of Sciences, Shiraz University, Shiraz, Iran; 2 Department of Fisheries, Faculty of Natural Resources, University of Guilan, Sowmeh Sara, Guilan, Iran

**Keywords:** Phylogenetic Relationship, Evolutionary History, *Capoeta* Gracilis, Caspian Sea basin

## Abstract

Traditionally, *Capoeta *populations from the southern Caspian Sea basin have been considered as *Capoeta capoeta gracilis*. Study on the phylogenetic relationship of *Capoeta *species using mitochondrial cytochrome *b *gene sequences show that *Capoeta *population from the southern Caspian Sea basin is distinct species and receive well support (posterior probability of 100%). Based on the tree topologies obtained from Bayesian and Maximum Likelihood methods, three main groups for the studied *Capoeta *were detected: Clade I) *Capoeta trutta *group (the Mesopotamian *Capoeta *group) including closely related taxa (e.g. *trutta*, *turani*, *barroisi*) characterized by having numerous irregular black spots on the dorsal half of the body. This clade was the sister group to all other *Capoeta *species and its separation occurred very early in evolution possess, so we considered it as O ld Evolutionary Group. Clade II) comprises highly diversified and widespread group, *Capoeta damascina *complex group (small scale capoeta group), the Anatolian-Iranian group (e.g. *banarescui*, *buhsei*, *damascina*, *saadii*), characterized by small scales and plain body (absence of irregular black spots on the dorsal half of the body, except in some juveniles) with signiﬁcantly later speciation event so called Young Evolutionary Group. Clade III) *Capoeta capoeta *complex group (large scale capoeta group, the Aralo-Caspian group) comprises very closely related taxa characterized by large scales and plain body (absence of irregular black spots on the dorsal half of the body) distributed in Aralo-Caspian water bodies (*capoeta*, *ekmekciae*, *heratensis*, *gracilis*, *sevangi*) that has been recently diverged and could be considered as Very Young Evolutionary Group.

## INTRODUCTION

The family Cyprinidae is the largest freshwater fish group in the world, including over 200 genera and 2947 species distributed throughout the world [1-3]. Cyprinids of the genus *Capoeta *(the local vernacular name "kapwaeti" used in Georgia and Azerbaikhan) are widely distributed through-out Western Asia from Anatolia to the Levant, Transcaucasia, the Tigris and Euphrates basins, most of Iran, Turkmenistan, Northern Afghanistan, and the upper reaches of the Amu- Darya and Syr-Darya drainages and generally occurs in lakes and streams with fast and slow- flowing waters [4-7]. The genus *Capoeta *is characterized by a compressed to rounded and moderately elongate body, small to moderately large scales (lateral line counts 37-99), scales at the anal fin base and anus not usually enlarged (sometimes variably enlarged as is the case with certain cyprinids), an inferior, transverse mouth, barbels in 1 or 2 pairs, dorsal fin short (usually 7-9 branched rays) with the last unbranched ray thickened and bearing serrations (serrations sometimes reduced to absent), anal fin short (usually 5 branched rays), gill rakers short, moderate to numerous, pharyngeal teeth in 3 rows with spoon- shaped and truncate tips, a very long and coiled gut (ca. 7-10 times body length), mostly of uniform colour, and a black peritoneum [8]. This genus contains about 23 species, of which 7 nominal species occur in Iran [8, 9] and most of them have been studied morphologically. However, the phylogenetic relationships of the genus *Capoeta *remained poorly studied until recent years when Levin et al [7] studied the complete mitochondrial cytochrome *b *sequences obtained from 20 species sampled from the majority of the range and 44 species of closely related barbs of the genera *Barbus *s. str. and *Luciobarbus. *According to Levin et al [7] *Capoeta *is a monophyletic clade nested within *Luciobarbus, *with origins in the Middle Miocene of a palaeo-Tigris-Euphrates basin. *Luciobarbus subquincunciatus *is the closest relative and is only found in the modern Tigris-Euphrates basin. The specialized algae scraping morphology appeared once within in the evolution of this genus.

Still the phylogenetic relationships of *Capoeta capoeta *complex group remain poorly studied until now. Traditionally, the Iranian *Capoeta capoeta *populations have been grouped in 4 subspecies: *Capoeta capoeta gracilis *(Keyserling, 1861) in the southern Caspian Sea and Urmia basins; *C. c. sevangi *de Filippi, 1865 in Aras river of south-western Caspian Sea basin; *C.c. intermedia *(Bianco & Banarescu, 1982) in the Persian Gulf basin (Helleh and Mond sub-basins) and *C. c. heratensis *(Keyserling, 1861) in the Tedzhen or Harirud River basin [4, 10 - 12]. Moreover, *C. capoeta capoeta *(Güldenstädt, 1773) also has been reported from Kura River basin (presumably in Iran also). As of date, three subspecies of *Capoeta c. capoeta*, *C. c. heratensis *and *C. c. sevangi *have got full species rank as *Capoeta capoeta*, *Capoeta heratensis *and *Capoeta sevangi *[13, 14, 7]. *Capoeta capoeta intermedia *was first considered as synonym of *Capoeta damascina *(Valenciennes, 1842) and now synonym of *Capoeta saadii *(Heckel, 1847) [see 7, 8]. However, still there is controversial debate about thesystematic position of *Capoeta capoeta gracilis. *Bianco & Banarescu [12] limit *C. c. gracilis *to basins between the Sefidrud River and the Atrak River (Caspian Sea basin) while *C. c. capoeta *is found in the Kura-Aras basin but Holčík & Jedlička [15] consider that the two subspecies *gracilis *and *heratensis *do not exist and the taxon *C. capoeta *exhibits clinal variation. Our main goals are to contribute to the understanding and exploring of the inner phylogeny of the genus *Capoeta *and to determine the systematic position of what known as *Capoeta c. gracilis *from Sefidrud River (Sefidrud River, southern Caspian Sea basin of Iran) using mitochondrial cytochrome *b *gene sequences.

## MATERIALS AND METHODS

**Figure 1:**
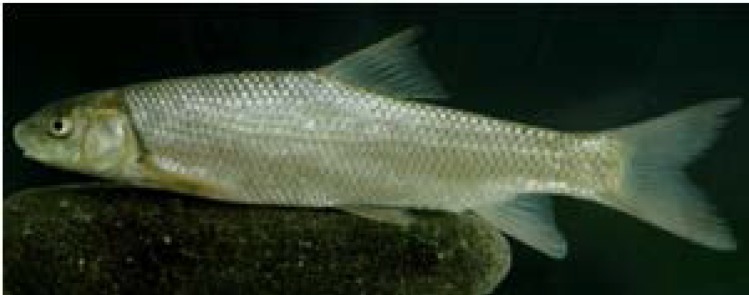
*Capoeta* *gracilis* from Sefidrud River, southern Caspian Sea basin

**Figure 2:**
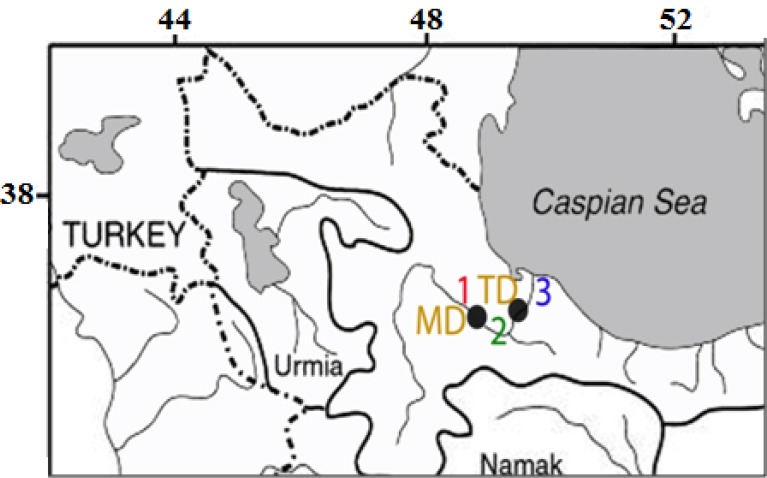
Location of sampling sites (1: Upstream of Manjil Dam, 2: Downstream of Manjil Dam, 3: Downstream of Tarik Dam, MD: Manjil Dam, TD: Tarik Da m).

**Sampling: **Specimens of *Capoeta gracilis *(Fig. 1) were collected from three sampling sites in Sefidrud River including upstream of Manjil dam (36°77′89″N, 49°15′25″E; station 1), downstream of Manjil dam that is upstream of Tarik dam (36°46′52.86″N, 49°61′18″E; station 2) and downstream of Tarik Dam (36°99′15″N, 49°57′71″E; station 3), in November 2013 using electrofishing (Fig. 2). The right pectoral fin of each fish was removed and preserved in 96% ethanol at the sampling sites and transported to laboratory.

**DNA extraction and amplification protocol: **Total genomic DNA was extracted according to phenol/chloroform procedures [16]. The partial mitochondrial cytochrome *b *gene was amplified by polymerase chain reaction (PCR) using two universal primers L14724 (5'-GTG ACT TGA AAA ACC ACC GTT G-3') and H15915 (5'-CAA CGA TCT CCG GTT TAG AAG AC-3') [17, 18]. Amplification was performed in a thermal cycler programmed as following: initial denaturation 94°C for 3 min, 35 cycles with denaturation at 94°C for 50 s, annealing 56°C for 45 s, extension 72°C for 1 min per cycle and followed by a final extension phase at 72°C for 5 Amplification products were checked by electrophoresis in 1% agarose gel in 0.5X TBE. Sequencing of the PCR product was conducted at Macrogen, Korea Laboratories with forward sequencing primer L14724 (5*P* *P* GAC TTG AAA AAC CAC CGT TG-3*P* *P*). New sequences were edited (see below) and are deposited in NCBI Genbank (www.ncbi.nlm.nih.gov) under accession numbers. New sequences from this study were combined with data from previous studies that obtained from the NCBI GenBank (see Table 1 for accession numbers).

**Sequence alignme nt and genetic analysis: **The *cytb *sequences were aligned and edited using BioEdit version 7.0.0 [19] and checked by eye for unexpected stop codons. The final aligned dataset included 900 bp for each taxon. Intergenetic distances were conducted using Mega 5 software and Kimura 2-parameter distance method. Bayesian inference (BI) was conducted to estimate phylogenetic relationships using MrBayes 3.1.2 [20] and using Markov-chain Monte Carlo tree searches for 2 million generations with a sampling frequency of 10. Four chains were used. Maximum Likelihood [21] were executed using Seaview4 [22] for examine the robustness the Bayesian results. In all models, phylogenetic trees were rooted using the outgroup species *Cyprinus carpio*, which belongs to the same family as the genus *Capoeta*.

## RESULTS

**Figure 3:**
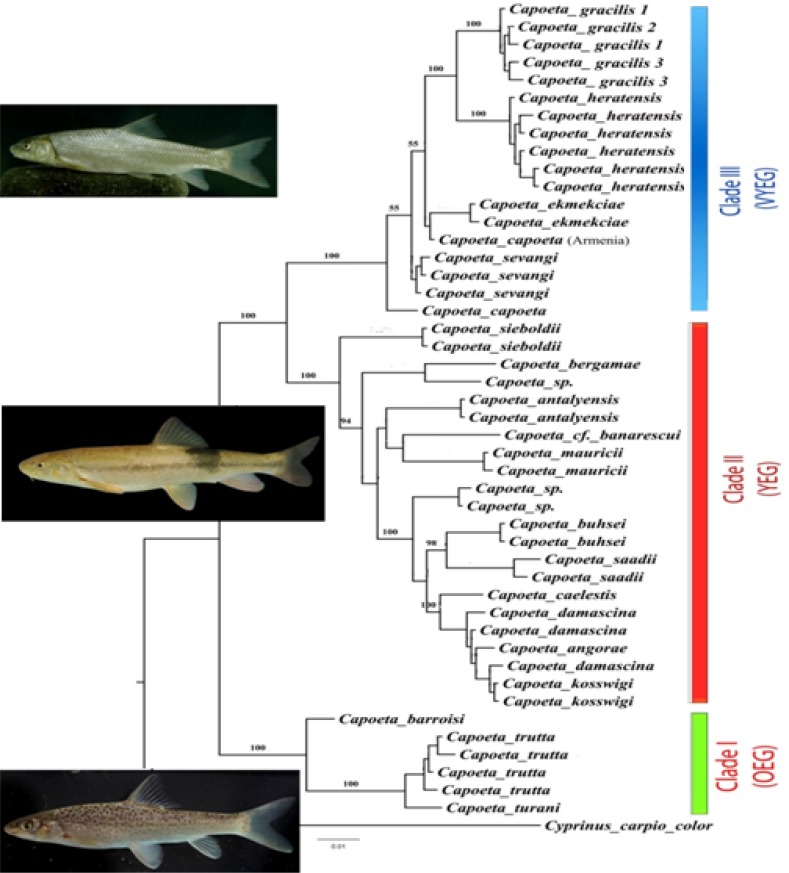
Bayesian in ference (BI) based on cytochrome *b *sequences to estimate phylogenetic relationships of *Capoeta*. Numbers on nodes indicate the posterior probability in percent based on 2000000 replicates

**Figure 4:**
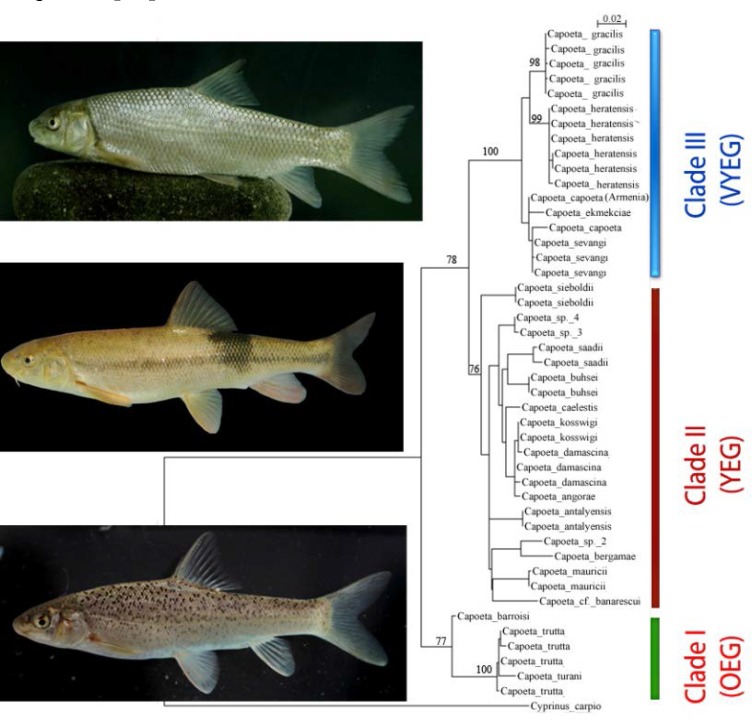
Maximum likelihood tree based on cyt *b *dataset of *Capoeta *Species. Bootstrap values are given above the nodes

Two phylogenetic approaches including Bayesian inference and maximum Likelihood also calculating the interspecific distances among species of *Capoeta *are given in Figures 3 and 4 and Table 1. The two different phylogenetic approaches produced almost similar tree topologies (Fig. 2, 3). There are three main clades in trees including I) *C. barroisi*, *C. trutta *and *C. turani*, II) *C. saadii*, *C. buhsei*, *C. koswigi*, *C. damascina*, *C. angorae*, *C. caelestis*, *C. maurici*, *C. antalyensis*, *C. bergamae*, *C. *cf. *banarescui *and *Capoeta *sp. and III) *C. sevangi*, *C. capoeta*, *C. heratensis*, *C. ekmekciae *and *C. gracilis*. In the Bayesian tree, *C. gracilis *specimens from three stations of Sefidrud River clustered together in one group as sister group with *C. heratensis *species in clade A. The topology of ML tree also was similar with that of Bayesian. The pairwise genetic divergence distances between the *C. gracilis *populations in Sefidrud River ranged from 0.1 to 0.2 whereas overall mean distance among all species of *Capoeta *was 0.055. Therefore for genetic inter-specific differences, compared with other *Capoeta *species, no distinct differences was found among *C. gracilis *populations in Sefidrud River (Table 2). The greatest genetic distance among *Capoeta *species was between *C. heratensis *and *C. turani *(10.1) and the smallest one between *C. angorae *and *C. damascina *(0.5).

**Table 1:** Species names, sampling localities, and GenBank Accession Numbers

Species	Accession Num.	Locality
*C. c. heratensis*	JF798319	Keltechinar River, Turkmenistan
*C. c. heratensis*	JF798318	Yanbash River, T urkmenistan
*C. c. heratensis*	JF798317	Yanbash River, T urkmenistan
*C. c. heratensis*	JF798316	Murgab River, Turkmenistan
*C. c. sevangii*	JF798301	Arpa River, Aras River basin, Armenia
*C. c. sevangii*	JF798302	Arpa River, Aras River basin, Armenia
*C. c. sevangii*	JF798299	Uraget River, Hrazdan, Aras River basin, Armenia
*C. sieboldii*	JF798330	Kelkit Cayi River, Black Sea basin, T urkey
*C. sieboldii*	JF798329	Kizilirmak River, Black Sea basin, Turkey
*C. caelestis*	JF798336	Ilica stream, Gulf of Antalya, Mediterranean Sea basin,Turkey
*C. turani*	JF798335	Çatkit River, Mediterranean Sea basin, Turkey
*C. trutta*	JF798334	Dez River, Rud e karun basin, Iran
*C. trutta*	JF798333	Sultansuyu River, Euphrates basin, T urkey
*C. trutta*	JF798332	Gelal River, Ab e Seymareh basin, Iran
*C. mauricii*	JF798325	Eflatum spring, Beysehir Lake basin, Turkey
*C. mauricii*	JF798324	Sarioz stream, Beysehir Lake basin, Turkey
*C. kosswigi*	JF798320	Deli Cayi River, Van Lake basin, Turkey
*C. kosswigi*	JF798321	Deli Cayi River, Van Lake basin, Turkey
*C. buhsei*	JF798283	Taghra Rud stream, Namak basin, Iran
*C. bergamae*	JF798282	Bakacak stream, Marmara Sea basin, T urkey
*C. antalyensis*	JF798269	Boga Cayi River, Mediterranean Sea basin, T urkey
*C. antalyensis*	JF798270	Boga Cayi River, Mediterranean Sea basin, T urkey
*C. angorae*	JF798268	Pozanti River, Mediterranean Sea basin, Turkey
*C. damascina*	JF798309	Karadut River, Euphrates basin, Turkey
*C. damascina*	JF798307	Yocalti River, Turkey
*C. damascina*	JF798308	Yocalti River, Turkey
*C. *cf. *banarescui*	JF798276	Kelkit Cayi River, Black Sea basin, T urkey
*C. saadii*	JF798326	Kor River, Kor basin, Iran
*C. saadii*	JF798327	Rodan River, Oman Gulf basin, Iran
*C. barroisi*	JF798279	Karasu River, Orontes basin, Turkey
*C. ekmeckiae*	GQ424027	Unknown
*Capoea sp.*	JF798340	Gelal River, Ab-e-Seymareh basin (Persian Gulf), Iran
*Capoea sp.*	JF798339	Gelal River, Ab-e-Seymareh basin (Persian Gulf), Iran
*Capoea sp.*	JF798337	Dalaman River, Aegean Sea basin, Turkey
*C. capoeta*	JF798289	Unknown
*C. capoeta*	AF145951	Sevan Lake, Armenia
*C. capoeta*	HM536882	unknown
*C. capoeta*	JF798331	Unknown
*C. gracilis* *	KT 982289	Sefidrud River, Caspian Basin, Upstream of Manjil Dam, Station 1
*C. gracilis* *	KT 982290	Sefidrud River, Caspian Basin, Upstream of Manjil Dam, Station 1
*C. gracilis* *	KT 982292	Sefidrud River, Caspian Basin, Downstream of Manjil Dam, Station 2
*C. gracilis*	KT 982288	Sefidrud River, Caspian Basin, Downstream of Tarik Dam, Station 3
*C. gracilis* *	KT 982291	Sefidrud River, Caspian Basin, Downstream of Tarik Dam, Station 3

* Sequences that have been obtained in this study.

## DISCUSSION

Traditionally, in Iranian freshwaters, six species (*Capoeta aculeata*, *Capoeta damascina*, *Capoeta buhsei*, *Capoeta fusca*, *Capoeta saadii *and *Capoeta trutta*) and seven subspecies (*Capoeta capoeta capoeta*, *Capoeta capoeta gracilis*, *Capoeta capoeta heratensis*, *Capoeta capoeta intermedia*, *Capoeta capoeta sevangi*, *Capoeta barroisi mandica *and *Capoeta barroisi persica*) represent the genus *Capoeta *[8,10,12]. The following taxa named from Iran have been regarded as synonyms of widely distributed species, *Capoeta damascina *(Valenciennes, 1842): *Scaphiodon amir *Heckel, 1849, *Scaphiodon niger *Heckel, 1849, *Scaphiodon chebisiensis *Keyserling, 1861, *Scaphiodon rostratus *Keyserling, 1861 and *Capoeta capoeta intermedia *Bianco & Bănărescu, 1982 (non *Capoeta intermedia *Temminck & Schlegel, 1846 = *Acheilognathus lanceolata *(Temminck & Schlegel, 1846) [12]. Recently, mitochondrial DNA (mtDNA) sequences have been used to explore and advance our understanding of species relationships especially the complex group such as *Capoeta*. A comparison of the different subspecies shows that several of them in fact are clearly distinct species.

**Table 2:** Pairwise comparisons of mean genetic distances of the studied *Capoeta *lineages based on the Kimura 2-parameter distance method (in present

**Species**	**1**		**2**		**3**		**4**		**5**		**6**		**7**		**8**		**9**		**10**		**11**		**12**		**13**		**14**		**15**		**16**		**17**	
*C. gracilis_*1 (1)																																		
*C. gracilis_*2 (2)	0.1																																	
*C. gracilis_*3 (3)	0.2		0.2																															
*C. heratensis *(4)	2.3		2.2		2.4																													
*C. sevangi *(5)	2.1		2		2.2		2.4																											
*C. capoeta *(6)	2.4		2.4		2.5		2.7		0.6																									
*C. ekmekciae *(7)	2.8		2.7		2.9		3.1		1.2		1.5																							
*C. turani *(8)	9.6		9.5		9.7		10.1		8.2		8.4		9.4																					
*C. trutta *(9)	9.4		9.3		9.5		9.5		8		8.3		9.2		1.2																			
*C. barroisi *(10)	7.2		7.1		7.3		7.6		6		6.2		7.1		3.6		3.1																	
*C. saadii *(11)	6.7		6.6		6.8		6.9		6.1		5.8		6.9		8.5		8.3		5.5															
*C. sieboldii *(12)	6.7		6.6		6.8		6.1		5.7		5.6		6.6		8.2		8.5		6.3		4.9													
*C. caelestis *(13)	6.3		6.2		6.4		5.6		5.4		5.1		6.2		8.2		8.2		5.3		3		3.4											
*C. buhsei *(14)	6.5		6.5		6.7		6.3		6.1		5.8		7		9.5		9.6		6.2		3		4.4		2.6									
*C. kosswigi *(15)	6.3		6.2		6.4		6.3		5.6		5.2		6.5		8.6		8.6		5.2		3		3.7		1.6		2.6							
*C. mauricii *(16)	6.9		6.8		7		7.2		5.8		5.8		6.7		9.2		8.9		7.2		4.7		5		4.1		4.1		4.1					
*C. damascina *(17)	6.3		6.3		6.5		6.4		5.7		5.3		6.5		8.5		8.6		5.2		3.1		3.7		1.7		2.6		0.3		4			
*C. bergamae *(18)	7.6		7.5		7.7		7.6		5.9		5.8		6.7		9.4		9.5		7.4		4.5		5.2		4.8		5.3		4.8		4.5		4.9	
*C. *cf. *banarescui *(19)			7		7		7.2		7		6.3		6.3		6.7		9.9		9.7		7.4		5.1		4.9		4.1		4.3		4.4		4.1		4.6	
*C. angorae *(20)			6.4		6.3		6.5		6.5		5.5		5.2		6.4		8.4		8.5		5.1		3		3.8		1.8		0.5		4		0.6		2.7	
*C.antalyensis *(21)			6.5		6.5		6.7		6.3		5.5		5.5		6.3		8.8		9.2		6.8		4.7		4.3		3.7		3.5		4		3.4		3.7	
*Cyprinus carpio *(22)			15.2		15.1		15.1		15		14.4		14.1		15.1		14.7		15.1		14.6		15.6		15.9		15.4		15.1		15.4		15		16	

Based on the tree topologies, three main groups were detected: Clade I), *Capoeta trutta *complex group (spotted capoeta group or the Mesopotamian capoeta group (*trutta*, *turani *and *barroisi*), Clade II) *Capoeta damascina *complex group (small scale capoeta, the Anatolian-Iranian group (*buhsei*, *damascina*, *saadii*), and Clade III) *Capoeta capoeta *complex group (large scale capoeta group, the Aralo-Caspian group (*Capoeta*, *ekmekciae*, *heratensis*, *gracilis*, *sevangi*).

The most diverged clade, the *Capoeta trutta *group, included closely related taxa such as *C. trutta *(Tigris, Euphrates and Orontes drainages), *C. turani *(Seyhan drainage, southern Anatolia) and *C. barroisi *(Tigris and Euphrates drainages) characterized by having numerous irregular black spots on the dorsal half of the body or completely flank and fin. Within this clade, *C. barroisi *is sister species to *C. trutta *+ *C. turani *and splitted earlier but good resolution was found for *C. trutta *and *C. turani *using the studied nucleotide sequences of *cytb*. This clade was the sister group to all other studied *Capoeta *species (Fig 3,4). According to Levin et al [7] its separation occurred in the Middle Miocene approximately 12.6 MYA. Here, we named it as O ld Evolutionary Group, OEG (see Figs 3,4). *Capoeta mandica *(Persian Gulf basin), *C. erhani *(Seyhan drainage, southern Anatolia) and *C. pestai *(Egirdir and Beysehir Lakes, Turkey) with irregular black spots on the dorsal half of the body also belong to the same species group. It was already hypothesized that *C. barroisi*, *C. pestai *and *C. trutta *are closely related species [23].

As, clearly demonstrated in Fig 3,4, another divergent event splited the rest of studied *Capoeta *lineage into two distinct groups namely *Capoeta damascina *complex group (CDCG) and *Capoeta capoeta *complex group (CACG). It has been estimated that, this event has happened about 9.1 MYA; 95% CI: 6.4–10.9) in the Tortonian period [7]. The most diversed group is small scale *Capoeta *(*Capoeta damascina *complex group) characterized by small scales and plain body (absence of irregular black spots on the dorsal half of the body) encompasses many species occupying the majority of *Capoeta*’s range, including Anatolia, the Zagros Mountains, Mesopotamia and the Iranian plateau (e.g., Namak, Kor, Esfahan, Hormuz, Makran, Sirjan, Kavir basins). *Capoeta damascina*, *Capoeta banarescui*, *Capoeta buhsei*, *Capoeta sieboldi*, *Capoeta saadii*, *Capoeta caelestis*, *Capoeta angorae*, *Capoeta kosswigi*, *Capoeta antalyensis*, *Capoeta bergamae*, *Capoeta mauricii *and some undescribed species (Figures 3, 4) are included in this clade. As this clade diverged very later than *Capoeta trutta *complex group, it is called Young Evolutionary Group (YEG). Within this clade, *Capoeta sieboldi*, a species with a single pair of barbells, an arched mouth and pleated lips found in Coruh drainage of northeastern Anatolia is sister species to the other *Capoeta *species and split off earlier (Fig 3,4). It is well in agreement with Levin et al. [7]. Within the clade III, (*Capoeta capoeta *complex group or large scale capoeta), there are very closely related taxa characterized by large scales and plain body (absence of irregular black spots on the dorsal half of the body) distributed in Aralo-Caspian water bodies (e.g., Kura and Aras river drainages, Lake Sevan drainages and many rivers from Sefidrud to Atrak) in Caspian Sea basin. For a long time, members of this group have been considered as different distinct subspecies including *C. c. sevangi*, *C. c. capoeta*, *C.c. gracilis *and *C.c. heratensis *[4, 5, 8, 10 - 12, 23, 24]. The *Cytb *sequences data does not corroborate the use of the classic subspecies nomenclature for *C. c. sevangi*, *C. c. capoeta *and *C.c. gracilis *and *C.c. heratensis*, but supports the use of species nomenclature for *C. sevangi*, *C. capoeta*, *C. gracilis *and *C. heratensis*. The clade III was formed by two recently diverged subgroups (Very Young Evolutionary Group, VYEG) approximately 2.6 MYA based on Levin et al. [7]. Based on the maximum Likelihood tree (Fig4), the first subgroup comprised *C. sevangi*, *C. capoeta *and *C. ekmekciae *which are widespread in the Kura and Aras rivers and Lake Sevan drainages (western Caspian Sea basin) but their interrelationships are not well resolved. The second subgroup comprised a well-supported subgroup of species (bootstrap values of 98% to 99%) that are distributed in the central and eastern parts of South Caspian Sea basin from Sefidrud River to Atrak River (*C. gracilis*) and also in the Tedzhen or Harirud River basin (*C. heratensis*). However, in the tree topology obtained from the Bayesian inference (BI), *Capoeta capoeta *is sister to all the other species of the clade III (Figure 3). In this tree, *C. gracilis *is sister to *C. heratensis *and receive well support (posterior probability of 100 %) and thus should be considered as two distinct species.

The detected pairwise sequence divergence between the studied species of the genus *Capoeta *lineage ranged from 0.5 to 10.1. This phylogenetic divergence was comparable to, or greater than that seen between some species of Teleostei [6, 14, 25 - 27]. In the present study the phylogenetic divergence between *C. gracilis *any other *Capoeta *species in *Capoeta capoeta *complex group is more than 2 (Table 2) and thus could be considered as a distinct species.The phylogenetic divergence among the three populations of *C. gracilis *in up and downstream of Manjil and Tarik dams in Sefidrud River was low 0.1 to 0.2 (Table 2) all clustered together in one group (Figure 3, 4). However, to study the genetic structures of three populations and understanding the effect of dams, using more specimens and other markers (e.g., microsatellite and D- loop) are suggested.

The results of this study show that *Capoeta *population from the southern Caspian Sea basin is distinct species (*Capoeta gracilis*) and is sister to *Capoeta heratensis *from the Tedzhen or Harirud River basin and receive well support (posterior probability of 100 %). The phylogenetic organization of *Capoeta *is composed of three main groups of *Capoeta trutta *complex group (spotted capoeta group), *Capoeta damascina *complex group (small scale capoeta) and *Capoeta capoeta *complex group (large scale capoeta group) with different evolutionary rates.
